# Phase transition, structural stability and electrical properties of V or Mn doped ZnSe composites under high pressure

**DOI:** 10.1038/s41598-025-89795-3

**Published:** 2025-02-12

**Authors:** Tao Liu, Yuxuan Huang, Shixia Wang, Yalin Wang, Ping Cheng, Jia Wu

**Affiliations:** 1 Department of Chemistry, University of Shanghai for Science and Technology, Shanghai, 200093 People’s Republic of China; 2 State Key Laboratory of Petroleum Resources and Prospecting, China University of Petroleum (Beijing), Beijing, 102249 People’s Republic of China

**Keywords:** V/Mn:ZnSe, First principles calculations, Phase transition pressure, Electrical properties, Magnetic properties, Nanoscience and technology, Chemistry, Electrochemistry, Inorganic chemistry, Theoretical chemistry

## Abstract

The structural parameters and enthalpies of pure ZnSe and different concentrations of V/Mn:ZnSe at high pressures were calculated using the first principles calculation method based on density functional theory. The lattice constants and bond lengths of all the systems decrease under pressure, and the respective phase transition pressures are obtained from the enthalpy-pressure relationship curves, which show that V/Mn elemental doping reduces the phase transition pressure of ZnSe, and the phase transition pressure further decreases with the increase of the doping concentration. The doping formation energies and the elastic constant criterion at atmospheric and high pressures confirm the structural stability of all the systems within the pressures of this study, and the pugh ratio confirms that they are all ductile structures.The results of electrical properties study show that at atmospheric pressure, all V:ZnSe systems have metallic properties, and a metal to semiconductor transition occurs at high pressure when the doping concentration is 12.5%. However, the Mn:ZnSe systems are semiconductors at both atmospheric and high pressures. Pressure significantly influences the degeneracy and position of the impurity bands: the impurity bands of V:ZnSe move toward higher energy under pressure, while the impurity bands of the Mn:ZnSe system shift toward lower energy. The element doping concentration also affects the d orbital degeneracy of V/Mn:ZnSe under atmospheric pressure, the degeneracy of V/Mn-d orbital decreases with the increase in doping concentration. Under high pressure, the degeneracy of the V/Mn-d orbital decreases further when the doping concentration is 3.13%, but the degeneracy is enhanced when the doping concentration is 6.25 and 12.5%. Doping can effectively change the phase transition pressure of ZnSe, and the pressure can effectively modulate the properties of this material.

## Introduction

II-VI group semiconductor materials have been widely used in many fields, such as optoelectronic devices^1–3^, in recent years due to their novel electrical and optical properties. ZnSe, as an important direct bandgap II-VI group semiconductor material, presents a zinc blende (ZB) crystal structure at room temperature and normal pressure. Due to its large forbidden bandwidth, electron confinement energy, and other characteristics, it is an ideal material for the manufacture of optoelectronic devices and blue light emitting diodes with good wide bandgap and wide wavelength transmission performance, as well as quantum well devices^4,5^.

High pressure has been recognized as one of the most important methods for discovering and probing the structure and properties of semiconductor materials, affecting the material's microstructure, electrostatic interactions between atoms, electron orbitals, and chemical bonding^6–8^. Since Piermarini and Block^9^ discovered in 1975 that ZnSe undergoes phase transition under high pressure, ZnSe crystal structure phase transition has become one of the focuses of experimental and theoretical research. Experimentally, Karzel et al.^10^, Lin et al.^11^ and Shigeaki Ono^12^ found that the transition pressure of ZnSe from ZB structure to RS structure is 13.5, 14.4 and 13.3GPa, respectively. In terms of theoretical predictions, Smelyansky et al.^13^, Yao^14^, Ferahtia et al.^15^ and Deng et al.^4^ used different methods and computational software to predict the theoretical transition pressures for the phase transition of ZnSe from the ZB structure to the RS structure as 16.9, 12.6, 13.3 and 14.09 GPa, respectively. In addition, it is well known that doping can effectively change the properties of materials. Some studies have found that doping with transition metals can impart novel properties to certain materials. For example, lin et al.^16^ explored the nature properties of 3d transition metal doped GaAs and GaP by theoretical calculations and found that the doping of V, Cr and Mn causes the system to appear in a ferromagnetic state. Cao et al.^17^ also explored the influence of transition metals V, Cr and Mn doping on the properties of MoS_2_ based on the first-principles method. They found that the system does not exhibit magnetic properties when doped with Cr, whereas both V and Mn doping can induce magnetic properties. Suo et al.^18^ investigated the electronic structure of V, Cr and Mn doped CdS using theoretical calculations. The results show that doping with transition metals V, Cr, and Mn can effectively enhance the electrical conductivity of CdS, and the band gap decreases with increasing atomic number of the doping elements. Lin et al.^19^ investigated the electronic structure of V, Cr and Mn doped ScN monolayer using theoretical calculations. They found that this doping can effectively enhance the electrical conductivity of the ScN monolayer, but the band gap increases gradually with the atomic number of the doping elements. It is not difficult to observe that transition metal doping brings about interesting changes in material properties. At present, the effect of Cr doping on the phase transition pressure of ZnSe, as well as its influence on properties under atmospheric and high pressures, has been investigated in some detail^4^. Doping with Cr can effectively reduce the phase transition pressure of ZnSe and alter its electrical and optical properties. Moreover, high pressure can further modulate these electrical and optical properties. However, the effects of V and Mn doping on the phase transition pressure and properties of ZnSe have not been extensively studied. It is speculated that doping may lead to changes in the structural stability and properties of ZnSe under both atmospheric and high pressures.

Therefore, in this study, we adopted the first principles calculation method based on density functional theory to systematically investigate the influence of different concentrations of V or Mn doping on the phase transition pressure of ZnSe. We also discussed the influence of pressure on structural parameters such as bond length and lattice constant of ZB-structured pure ZnSe and V/Mn:ZnSe materials. The mechanical properties of ZB-structured pure ZnSe and V/Mn doped systems were calculated at atmospheric and high pressures to assess their mechanical stability under different pressures. Finally, the effect of pressure on the electronic structure and magnetic properties of both pure ZnSe and V/Mn:ZnSe materials was investigated.

## Materials and methods

**Fig. 1:**
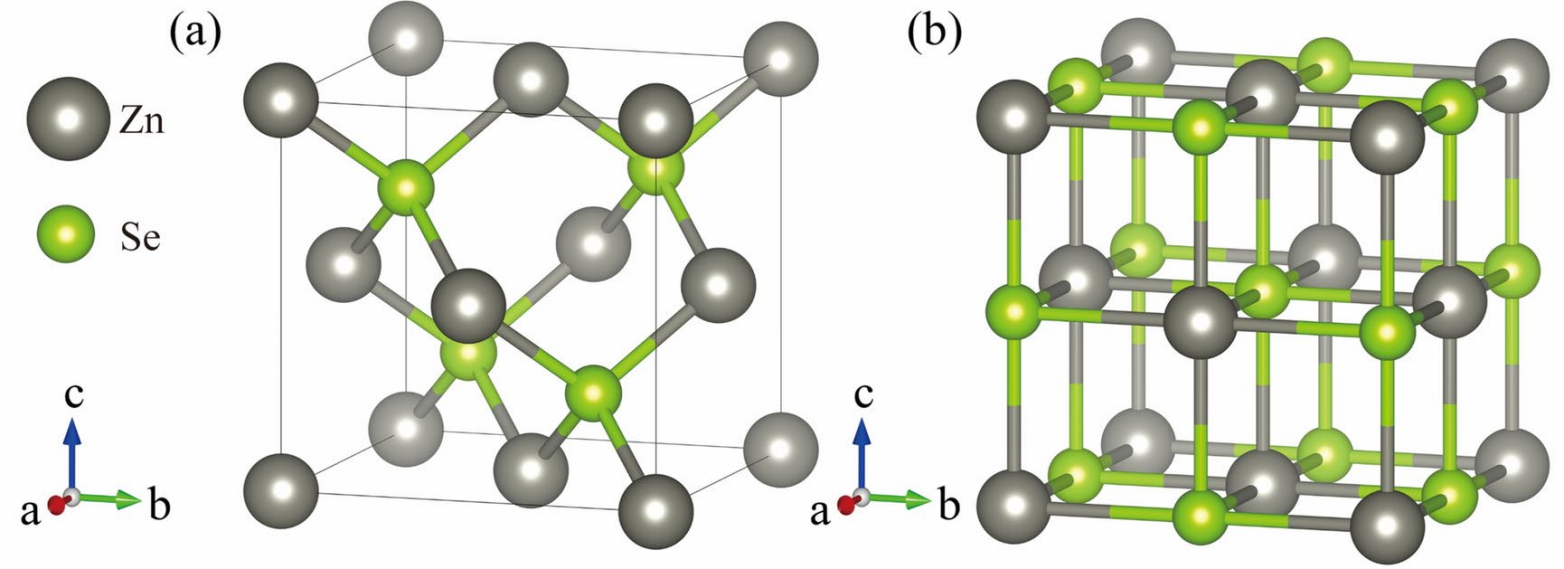
Schematic representation of the original crystal structure of ZnSe (By VESTA) (**a**) ZB phase; (**b**) RS phase.

**Fig. 2:**
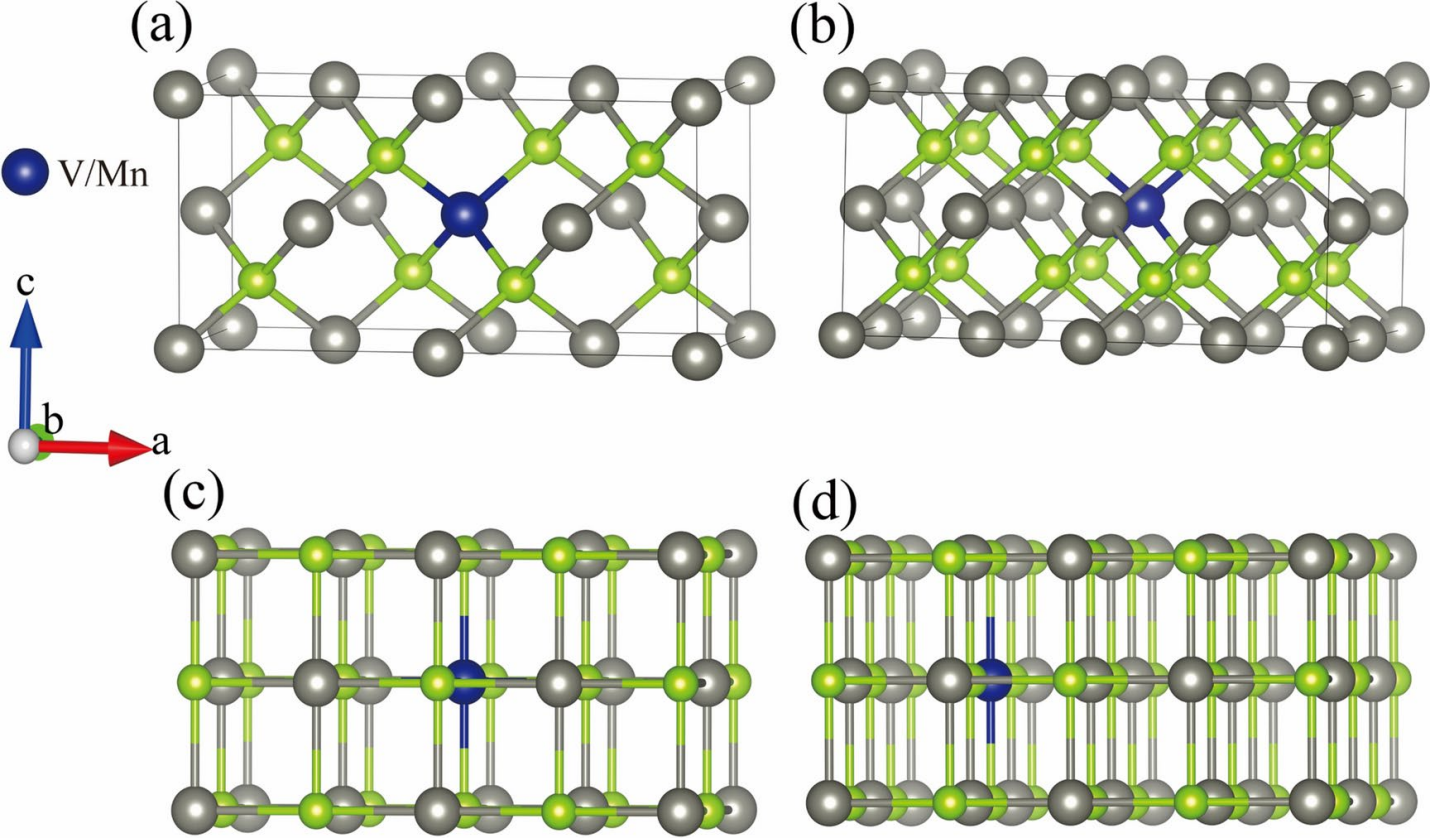
Schematic representation of ZnSe supercell doping system (By VESTA) (**a**) ZB structure 2 × 1 × 1 supercell; (**b**) ZB structure 2 × 2 × 1 supercell; (**c**) RS structure2 × 1 × 1 supercell; (**d**) RS structure 2 × 2 × 1 supercell.

Figure 1 shows the original structure diagram of the ZnSe crystal in sphalerite structure (ZB) and rock salt structure (RS), where the ZB phase space has space group $F\overline{4}3m$ and the RS phase has space group $Fm\overline{3}m$. Supercells of 2 × 1 × 1 and 2 × 2 × 1 for the original structure yielded models containing 16 and 32 atoms, as shown in (Fig. 2). According to the experience of previous studies, the positions of Zn atoms in the ZnSe structure are equivalent in three-dimensional space, and the formation energies at different positions are nearly identical. Therefore, in both the original structure and the supercell, we randomly selected a Zn atom position to replace a V or Mn atom, with atom substitution doping concentrations of 3.13, 6.25, and 12.5%, respectively.

In this paper, based on the density functional theory (DFT) plane wave pseudopotential method, calculations were carried out using the VASP software^20^. Using projected Augmentation wave (PAW), exchange dependent interactions are treated in generalized gradient approximation (GGA) in the form proposed by Perdew, Burke, and Ernzerhof (PBE)^21^. The plane wave truncation energy used in all calculations is 450 eV, with total energy and force convergence criteria set at 1 × 10^–7^ eV and 0.001 eV/Å, respectively. A Monkhorst–Pack grid with a density of 2Π × 0.04 Å⁻^1^ was used to sample the Brillouin zone during structural optimization. To ensure the accuracy of the calculated electronic structures under different pressures, we employed a Monkhorst–Pack grid with a density of 2Π × 0.03 Å⁻^1^ to sample the optimized structures of both pure ZnSe and V/Mn:ZnSe materials. Based on the VASPKIT software package for processing the calculation results^22^, its accuracy and validity have been confirmed by many previous studies^23–25^.

## Results and discussion

### Phase transition pressure

According to the thermodynamic stability theory, the phase with the lower free energy corresponds to the more stable phase. In order to investigate the effect of different concentrations of transition metal V and Mn doping on the stability of the ZnSe structure, it is necessary to calculate the free energies of the two structures, ZB and RS, at different pressures. It is well known that the free energy is defined as $\text{G}=\text{E}+\text{PV}-\text{TS}$,and since the calculations are carried out under a temperature condition of 0 K, the last term TS can be neglected. The free energy G can be simplified to enthalpy, and the corresponding enthalpy equation is^4,26^:

1 $$H=E+PV$$

The enthalpies of the two different structures of ZnSe will be the same when the phase transition occurs, as shown in (Supplementary Fig. 1). The results show that pure ZnSe will transition from the ZB structure to the RS structure at 14.15GPa, which is consistent with the results of previous studies^4,13–15,26,27^. The addition of V reduced the phase transition pressures to 9.97 GPa (3.13%), 7.74 GPa (6.25%), and 4.36 GPa (12.5%), respectively, while the doping of Mn reduced the phase transition pressures to 11.07 GPa (3.13%), 9.72 GPa (6.25%), and 8.30 GPa (12.5%), respectively. The introduction of V/Mn impurities lowers the phase transition pressure of ZnSe, and the higher the doping concentration, the lower the phase transition pressure. This is because V/Mn doping reduces the free energy of the ZnSe system, and the higher the doping concentration, the lower the free energy. Previous researchers studying Fe-doped ZnS and CeO_2_ also found similar results^27,28^. V doping makes the phase transition pressure change more sensitive compared to Mn doping. The possible reason is that the outermost electron configuration of V^2^⁺ is d^3^, while that of Mn^2^⁺ is d^5^, which is a more stable half-filled state. Under pressure, Mn^2^⁺ is less likely to undergo charge rearrangement, leading to the phase transition pressure change being less sensitive in Mn-doped ZnSe compared to V-doped ZnSe.

**Fig. 3:**
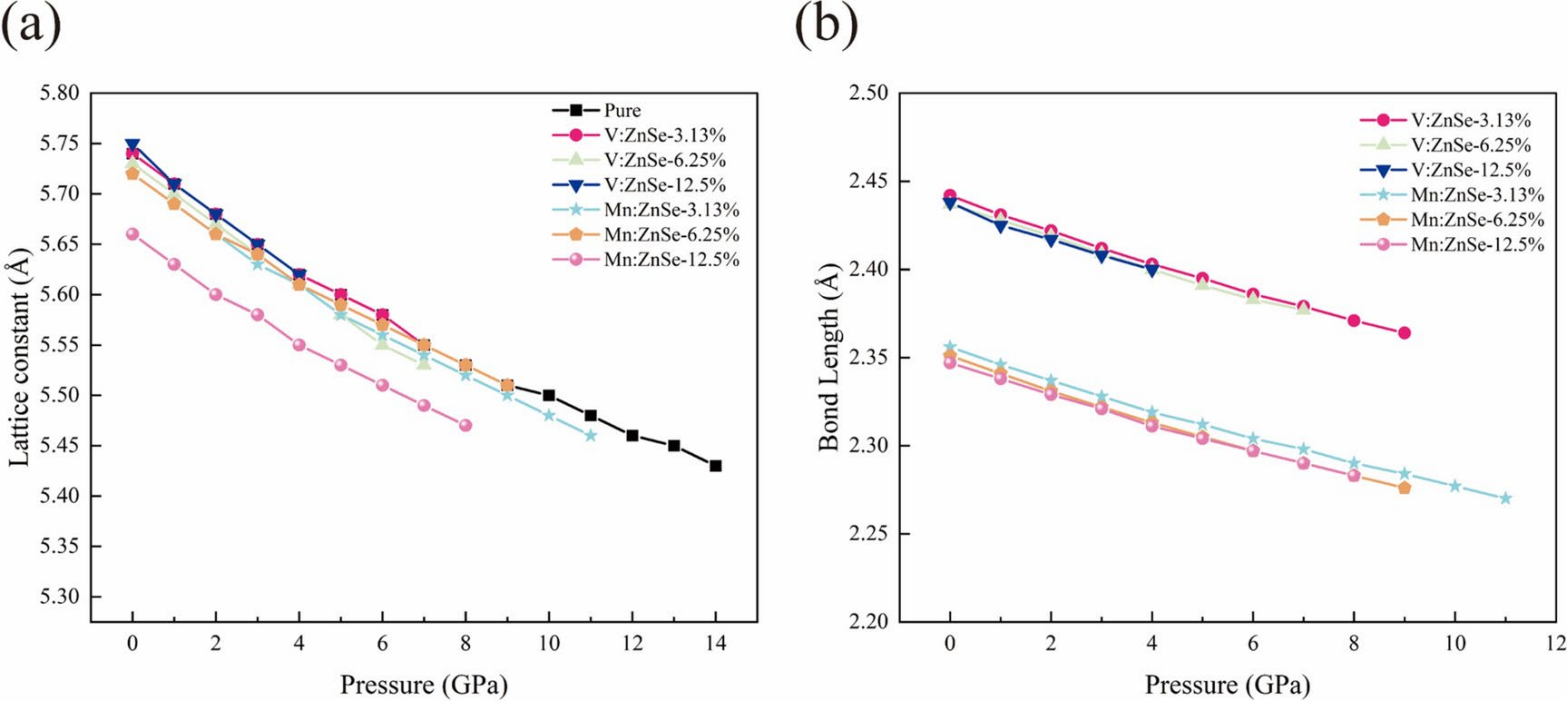
(**a**) Lattice constants versus pressure for pure ZnSe and V/Mn:ZnSe (**b**) V/Mn-Se bond length versus pressure for V/Mn:ZnSe.

Experimentally the elemental doping of ZnSe materials is usually done using crystals with ZB-type structures, so in the following we further investigate the structure and properties of ZB-structured pure ZnSe and V/Mn:ZnSe materials under high pressure. The optimized lattice constant of pure ZnSe for the ZB structure is a = b = c = 5.74 Å, which is in better agreement with previous calculations^15,26^, shows that our calculation method is reasonable. As shown in Fig. 3a, the lattice constants of both the ZB-structured pure ZnSe and V/Mn:ZnSe materials decrease linearly with increasing applied hydrostatic pressure. This tendency arises because the bond lengths between the Zn and Se atoms shorten as the pressure increases^4^. It is remarkable that the optimized lattice constants of V/Mn-doped ZnSe at atmospheric pressure are almost the same as those of pure ZnSe, we consider that this is mainly due to the similarity of the radius of the Zn atom (125 pm) with that of the V/Mn atom (122/117 pm). In Fig. 3b, the bond lengths of V/Mn:ZnSe are investigated, and the bond lengths of V-Se and Mn-Se at atmospheric pressure are nearly identical across all V or Mn doping concentrations. We observe that the bond lengths between the V/Mn impurity atoms and the Se atoms decrease with increasing external pressure. These changes in lattice constants and V/Mn-Se bond lengths will necessarily alter the properties of the material, such as phase transition pressure and electronic structure.

### Structural stability

The formation energy (E_f_, the formula is as follows^29^) and mechanical constants of the doping system were further calculated to determine the stability and feasibility of ZnSe doping:

2 $${E}_{f}={E}_{V/Mn:ZnSe}-{E}_{pure ZnSe}+{\mu }_{Zn}-{\mu }_{V/Mn}$$

where ${E}_{V/Mn:ZnSe}$ is the total energy of the system after substitution of a V/Mn atom for a doped Zn atom, ${E}_{pure ZnSe}$ is the energy of pure ZnSe before doping, ${\mu }_{V/Mn}$ is the chemical potential of the V/Mn atom, ${\mu }_{Zn}$ is the chemical potential energy of the Zn atom, and the value of ${\mu }_{Zn}{-\mu }_{V/Mn}$ is derived based on the calculations methods proposed by the previous authors^30^. The formation energies were calculated as −1.87, −1.90, and −1.93 eV when the V:ZnSe amounts were 3.13, 6.25, and 12.5%, respectively. While the formation energies for Mn:ZnSe amounts of 3.13, 6.25, and 12.5% were −1.15, −1.23, and −1.32 eV, respectively. Under high pressure, the formation energies of these systems were −1.73, −1.85, and −1.93 eV for V:ZnSe, and −1.09, −1.20, and −1.34 eV for Mn:ZnSe. The formation energies of these doping systems are all relatively small and negative, indicating that the doping process of these systems is exothermic and relatively stable, which is easier to achieve in experiments both under normal and high-pressure conditions.

The elastic constants of materials under hydrostatic conditions are important for predicting and understanding the response, strength, and mechanical stability of materials. To further investigate the mechanical properties of ZnSe and V/Mn:ZnSe structures under hydrostatic pressure, the second-order elastic constants, C_ij_, are calculated in this paper. The ZB structure of ZnSe belongs to the cubic crystal system with space group $F\overline{4}3m$. Due to symmetry relations, it exhibits three independent elastic constants: C_11_, C_12_, and C_44_. The elastic constants must satisfy the Born stability criterion under atmospheric pressure ito demonstrate the material's mechanical stability^31^:

3 $${C}_{11}>0,{C}_{44}>0,{C}_{11}>{C}_{12},{C}_{11}+2{C}_{12}>0$$

Under hydrostatic pressure, the criterion conditions for the mechanical stability of ZB structured ZnSe change^4,32,33^:

4 $${C}_{11}+2{C}_{12}+P>0,{C}_{44}-P>0,{C}_{11}-{C}_{12}-2P>0$$

Tables 1 and Table 2 we summarize the results of the elastic constants C_ij_ of pure ZnSe and different concentrations of V/Mn:ZnSe at atmospheric and high pressures, as well as the results of their mechanical stability criterion under hydrostatic pressure, and give the previous experimental data and the existing theoretical data for comparison. For pure ZnSe our calculated elastic constants (C_11_, C_12_ and C_44_) are in better agreement with those of Denget al.^4^ and Ferahtia et al.^15^ and the errors are within acceptable error limits. For the elemental doped systems, the effect of high pressure on C_44_ is minimized for all systems. The mechanical strength of ZB structure pure ZnSe and V/Mn:ZnSe is greater under high pressure compared to atmospheric pressure. Additionally, the elastic constants of all systems at atmospheric and high pressures are numerically in the relationship C_11_ > C_12_ > C_44_. All elastic constants satisfy the criterion of Eqs. (3) and (4), demonstrating that these materials are mechanically stable under both atmospheric and high pressures.

**Table 1:** Elastic constants of ZB-structured ZnSe and V/Mn doped ZnSe systems.

	Pressure(GPa)	C_11_(GPa)	C_12(_GPa)	C_44_ (GPa)
Pure ZnSe	0	80.98	45.08	37.43
	Theor.(0)	80.98^a^	45.87^a^	37.61^a^
		86.99^b^	53.37^b^	35.48^b^
	Expt.(0)	85.9^c^	50.6^c^	40.6^c^
	14	132.19	89.56	43.16
V:ZnSe-3.13%	0	75.70	46.20	34.57
	9	110.59	77.711	39.036
V:ZnSe-6.25%	0	72.09	43.40	33.49
	7	99.08	70.83	35.88
V:ZnSe-12.5%	0	66.89	46.84	25.96
	4	89.12	58.47	29.95
Mn:ZnSe-3.13%	0	78.24	45.67	32.57
	11	122.28	83.83	20.44
Mn:ZnSe-6.25%	0	80.22	43.57	29.59
	9	112.56	80.23	23.50
Mn:ZnSe-12.5%	0	74.15	49.41	26.56
	8	104.71	83.46	27.50

^a^Ref.^4^.

^b^Ref.^15^.

^c^Ref. ^34^.

**Table 2:** Results of mechanical stability criterion under high pressure.

	Pressure (GPa)	C_11_ + 2C_12_ + P (GPa)	C_44_-P (GPa)	C_11_-C_12_-2P (GPa)
Pure ZnSe	14	325.21	29.16	14.63
V:ZnSe-3.13%	9	275.012	30.036	14.879
V:ZnSe-6.25%	7	247.74	28.88	14.25
V:ZnSe-12.5%	4	210.06	25.95	22.65
Mn:ZnSe-3.13%	11	300.94	9.44	16.45
Mn:ZnSe-6.25%	9	282.02	14.50	14.33
Mn:ZnSe-12.5%	8	279.63	19.5	5.25

Based on the calculated independent elastic constants, the bulk modulus B, Young's modulus E, shear modulus G and B/G (pugh ratio) of the ZnSe system can be calculated. The bulk modulus B reflects the ability of the material to resist volume changes, the Young’s modulus E reflects the degree of rigidity of the material during the elastic deformation stage, the shear modulus G reflects the ability of the material to resist shear deformation, and the B/G (pugh ratio) evaluates the relative toughness and brittleness of the material. From Table 3, it is observed that the pugh ratios of the ZnSe systems are all greater than 1.75 in each cases, indicating that these structures exhibit ductility under both atmospheric and high pressures. At atmospheric pressure, all doped systems exhibit smaller B, E, and G values compared to the pure phase, indicating that V and Mn doping reduce the incompressibility, stiffness, and resistance to shear deformation of the ZnSe system. At high pressures, both the pure phase and V-doped systems exhibit improved B, E, and G values compared to those at atmospheric pressure, indicating that pressure enhances their incompressibility, stiffness, and resistance to shear deformation. However, the Mn-doped ZnSe systems show a special tendency to change, and their B-values are increased at high pressures, but the E and G values are decreased to some extent, which implies that the stiffness and the resistance to shear deformation of the Mn-doped ZnSe systems are reduced under pressure, except for the incompressibility, which is enhanced.

**Table 3:** Bulk modulus(B), Young’s modulus(E), hear modulus(G) and pugh ratio(B/G) at atmospheric and high pressures for each system.

	Pressure (GPa)	B (GPa)	E (GPa)	G (GPa)	B/G
Pure ZnSe	0	57.05	71.90	27.87	2.05
	14	103.77	88.32	32.52	3.19
V:ZnSe-3.13%	0	56.04	64.29	24.56	2.28
	9	88.67	75.00	27.59	3.21
V:ZnSe-6.25%	0	52.97	62.17	23.83	2.22
	7	80.25	67.18	24.69	3.25
V:ZnSe-12.5%	0	53.53	47.90	17.73	3.02
	4	68.69	61.79	22.89	3.00
Mn:ZnSe-3.13%	0	56.53	64.59	24.66	2.29
	11	96.95	55.98	19.94	4.85
Mn:ZnSe-6.25%	0	55.79	63.93	24.42	2.28
	9	91.01	56.50	20.23	4.50
Mn:ZnSe-12.5%	0	57.66	52.69	19.55	2.95
	8	90.54	52.71	18.78	4.82

### Electronic structure

**Fig. 4:**
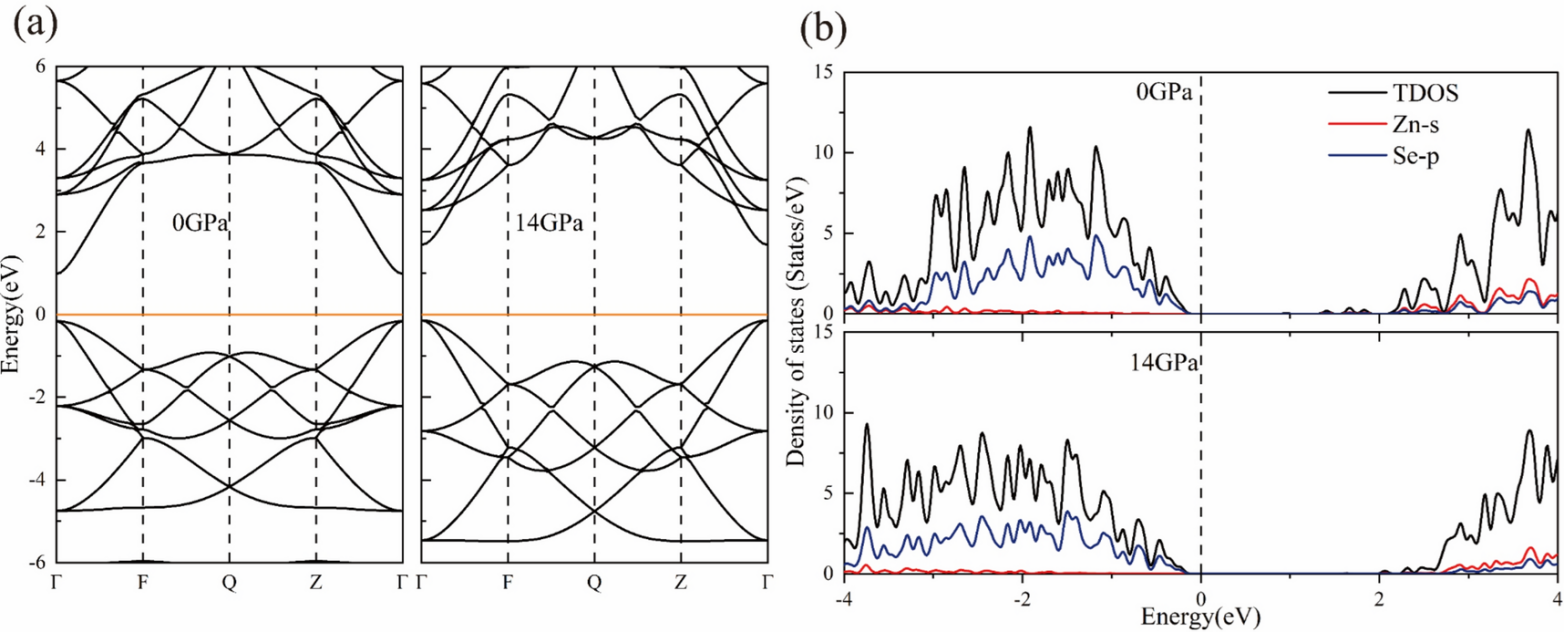
Electronic structure of ZB structure pure ZnSe at atmospheric and high pressure. (**a**) Energy bands; (**b**) Density of states.

**Fig. 5:**
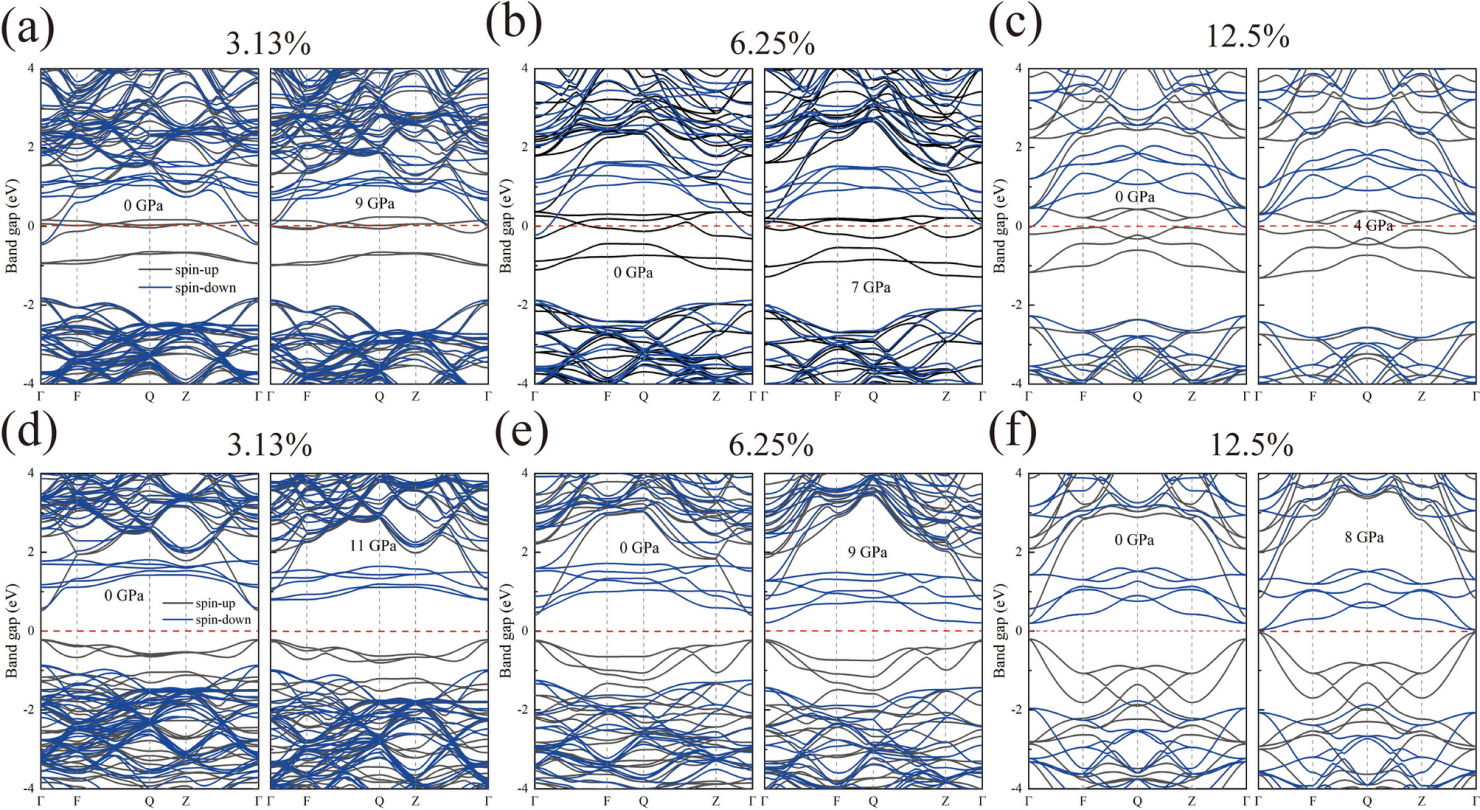
Energy bands of V/Mn: ZnSe system at atmospheric and high pressure. (**a**–**c**) V:ZnSe; (**d**–**f**) Mn:ZnSe.

In order to investigate the effect of high pressure on the electronic structure of ZB structured pure ZnSe and V/Mn:ZnSe materials, their energy band structures and densities of states at different pressures were calculated. It is well known that the inherent defects of the PBE functionals lead to an underestimation of the bandgap width^35^. However, this does not affect the comparison of the trends in electronic structure before and after doping and under pressure in this paper. From Fig. 4a, it can be observed that pure ZnSe is a direct bandgap semiconductor with a bandgap of 1.14 eV, where the minimum value of the conduction band (CBM) and the maximum value of the valence band (VBM) are located at the Γ point. Under high pressure, pure ZnSe remains a direct bandgap semiconductor, but the conduction band shifts towards higher energy directions, resulting in an increased bandgap. At atmospheric pressure, when a V or Mn atom replaces a Zn atom, doping causes impurity bands (IBs) to be created in the energy band structure, leading to a decrease in the bandgap after doping, which implies that the doping of the transition metal V/Mn facilitates the electron hopping. Among them, the IBs of the V:ZnSe system crosses the Fermi energy level, transforming it into a metal. In contrast, Mn:ZnSe system remains semiconducting, with the bandgap decreasing with increasing doping concentration. Under high pressure, the band gap of the V:ZnSe system with 12.5% doping concentration increases and transforms from metallic to semiconducting properties, which we speculate may be due to the fact that the high pressure induces microscopic defects or changes in stress in the material, which may lead to the transition from metallic to semiconducting at this doping concentration near the point of phase transition. while the metallic properties are maintained at both 3.13% and 6.25% doping concentrations. For the Mn:ZnSe material, all systems maintain semiconducting properties at high pressure, as shown in (Fig. 5).

The degeneracy of IBs decreases for both V and Mn doping systems with increasing concentration of doping atoms under normal pressure. However, the degeneracy patterns become more complex under high pressure. For V/Mn:ZnSe, at a doping concentration of 3.13%, the degeneracy of IBs weakens under high pressure. However, at doping concentrations of 6.25 and 12.5%, the degeneracy of IBs increases under high pressure. At the same time, it is also observed that under the action of high pressure, the IBs of the V:ZnSe system always moves towards the high energy method, while the IBs of the Mn:ZnSe system moves towards the low energy direction. This indicates that the pressure can effectively regulate the degeneracy and position of the IBs in the energy band structure of transition metal doped ZnSe materials, thus changing the band gap width,and the corresponding IBs with different dopant atoms and doping ratios have different trends with pressure.

**Fig. 6:**
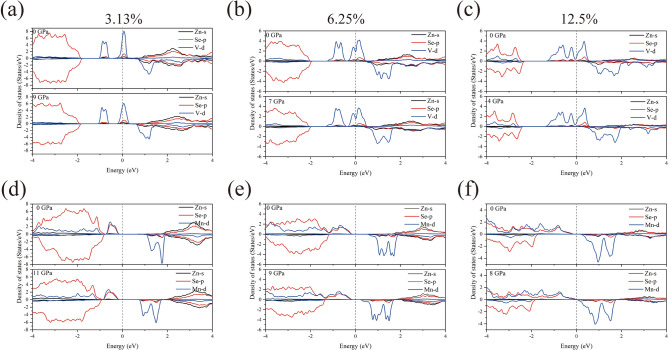
Density of states of V/Mn: ZnSe system. (**a**–**c**)V:ZnSe; (**d**–**f**) Mn:ZnSe.

Figure 4b and Fig. 6 show the density of states of pure ZnSe and V/Mn:ZnSe at atmospheric and high pressures. From Fig. 4b, we can intuitively find that the conduction band portion of pure ZB-structured ZnSe in the vicinity of the Fermi energy level under the pressures of 0 GPa and 14 GPa ZnSe is mainly contributed by the Zn-s and Se-p orbitals, while the valence band portion is mainly contributed by the Se-p orbital. In Fig. 6 we can find that the density of states near the Fermi energy level for any concentration of V/Mn:ZnSe material at atmospheric pressure are all mainly contributed by the d orbitals of impurity atoms. The orbital contributions to the valence and conduction band parts of all the systems do not change significantly under pressure.We speculate that this is because the optimized structure selected for the electronic structure calculation is subjected to a pressure lower than the phase transition pressure of ZnSe, which at this time is still stabilized in the ZB structure. After introducing impurity atoms, the energy bands and density of states for spin-up and spin-down exhibit asymmetric characteristics, indicating that the doped system is likely to possess magnetic properties. Related analysis can be found in the supplementary information.

**Fig. 7:**
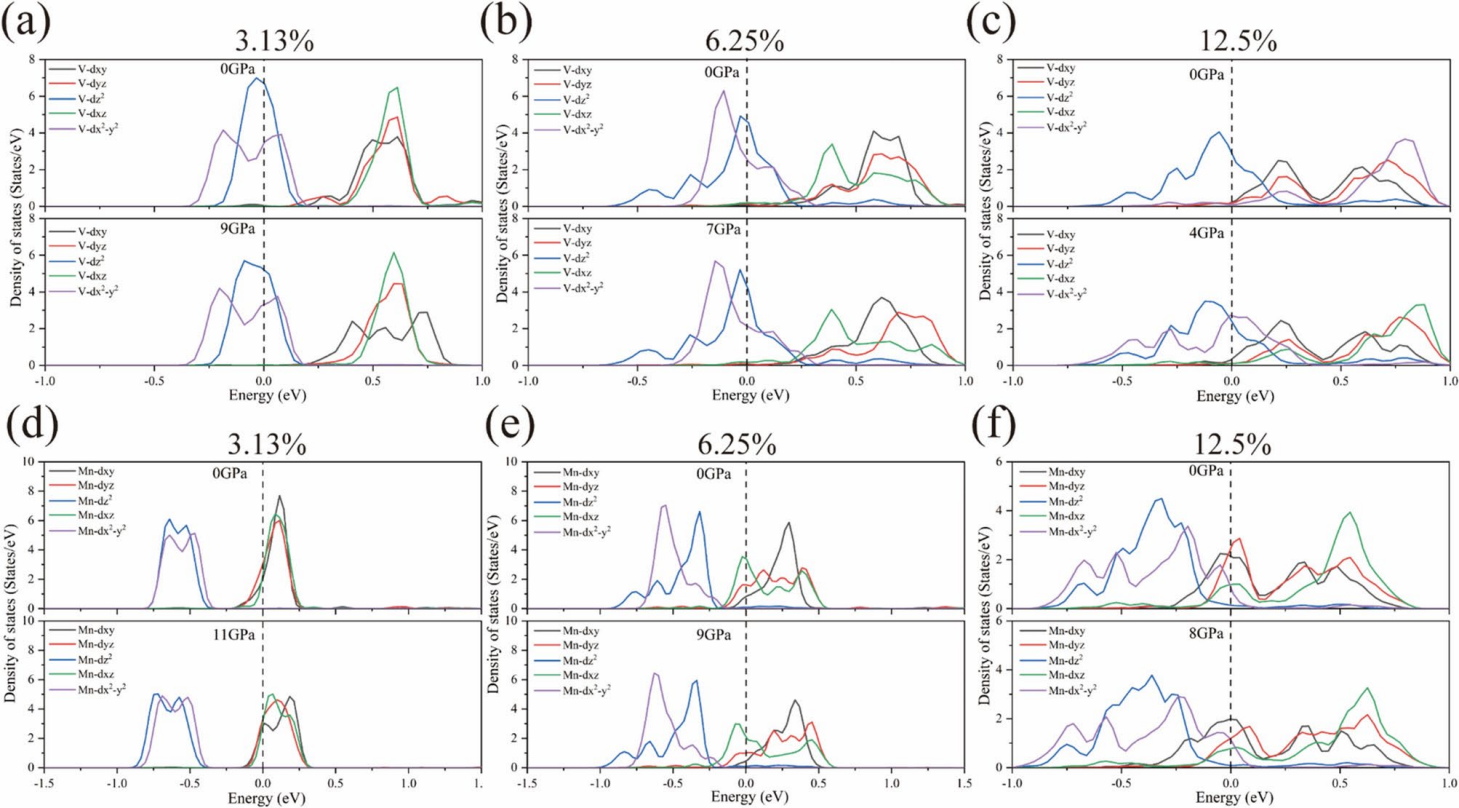
V/Mn-d orbital fractional density of states. (**a**–**c**) V:ZnSe; (**d**–**f**) Mn:ZnSe.

In order to study the effect of high pressure on IBs in detail, we also calculated the density of states in the d-orbitals of V/Mn-doped atoms at atmospheric and high pressures, as shown in (Fig. 7). When V/Mn atoms replace Zn atoms in ZnSe crystals, the atomic environment of the transition metal is tetrahedral symmetric. According to crystal field theory, in a tetrahedral field (T_d_), the five degenerate d orbitals can be split into two groups: the lower energy double degenerate orbitals E (d_x_^2^-_y_^2^, d_z_^2^), and the higher energy triple degenerate orbitals T_2_ (d_xy_, d_xz_, d_yz_). As can be seen from Fig. 7, the degeneracy of d orbitals of all impurity atoms decreases with the increase of doping concentration of V/Mn atoms under normal pressure. Under the action of high pressure, the degeneracy of d orbitals of systems with doping concentration of 3.13% weakens, while the degeneracy of d orbitals of systems with doping concentration of 6.25 and 12.5% is enhanced to some extent. The enhancement and weakening of these degeneracy will ultimately significantly affect the position of IBs, which is consistent with the results observed in the energy band.

## Conclusion

In summary, in this paper, the phase transition pressure, structural parameters, stability and electrical properties of the pure ZnSe and different concentrations of V/Mn-doped ZnSe are investigated based on the first-principles calculations. Based on the principle of enthalpy equivalence, we calculate that the phase transition pressure for the transition from the ZB structure to the RS structure of pure ZnSe is 14.15 GPa. For V/Mn:ZnSe, the phase transition pressure decreases with increasing doping concentration. The phase transition pressures for V:ZnSe-3.13%, V:ZnSe-6.25% and V:ZnSe-12.5% to undergo ZB structure to RS structure were 9.97 GPa, 7.74 GPa and 4.36 GPa, respectively, and those for Mn:ZnSe-3.13%, Mn:ZnSe-6.25% and Mn:ZnSe-12.5% to undergo phase transition pressures were 11.07 GPa, 9.72 GPa and 8.30 GPa, respectively. The extent of change in the phase transition pressure was greater for V:ZnSe at the same doping concentration. All calculated structural parameters, mechanical and electrical properties at high pressures in this paper correspond to pressures lower than the phase transition pressure, i.e., all structures maintain the ZB structure. The formation energies and elastic constants of the doped systems were obtained computationally, determining the stability of the structures at high pressures. Under pressure, the structural parameters of the systems (bond lengths and lattice constants) decrease linearly, and the bandgap of pure ZnSe increases with increasing pressure, maintaining the direct bandgap semiconductor properties at both atmospheric and high pressures, where the pressure causes the conduction band part of it to move toward the higher energy region. For the V: ZnSe systems, all have metallic properties at atmospheric pressure, and a metal to semiconductor transition occurs under pressure when the doping concentration is 12.5%. The Mn:ZnSe systems are semiconductors at both atmospheric and high pressures.Through the band structure of the doping system and the DOS of the V/Mn-d orbital, we find that the degeneracy of IBs decreases with the increase of the concentration of doping atoms at atmospheric pressure. The IBs degeneracy of V/Mn:ZnSe-3.13% is further weakened under high pressure, while the IBs degeneracy of V/Mn:ZnSe-6.25% and V/Mn:ZnSe-12.5% are enhanced. Pressure can significantly affect the location of IBs in these systems. The theoretical predictions of this study will provide guidance for subsequent experiments. The study of high-pressure phase transitions provides an important theoretical basis for the high-pressure synthesis of novel functional materials and the formation of compounds with special physical and chemical properties. High pressure can be seen as an effective means of regulating material properties.

## Supplementary Information

Supplementary Information.

## Data Availability

The datasets used and/or analysed during the current study available from the corresponding author on reasonable request.
