# Supplementary material for: Phase transition, structural stability and electrical properties of V or Mn doped ZnSe composites under high pressure
Source: Sci Rep. 2025 Feb 12;15:5227. doi: 10.1038/s41598-025-89795-3 (PMC11821951; doi:10.1038/s41598-025-89795-3)
Supplement: Supplementary file 1 — Supplementary Information. [file 41598_2025_89795_MOESM1_ESM.docx]

**Supplementary Information**

Phase transition, structural stability and electrical properties of V or Mn doped ZnSe composites under high pressure

Tao Liu,^a^ Yuxuan Huang, ^a^ Shixia Wang, ^a,*^ Yalin Wang,^a^ Ping Cheng ,^a^ Jia Wu^b^

*^a^* Department of Chemistry, University of Shanghai for Science and Technology, Shanghai 200093, P. R. China.

^b^ State Key Laboratory of Petroleum Resources and Prospecting, China University of Petroleum (Beijing), Beijing 102249, PR China.

*Corresponding author: E-mail: [wangshixia@usst.edu.cn](mailto:wangshixia@usst.edu.cn)


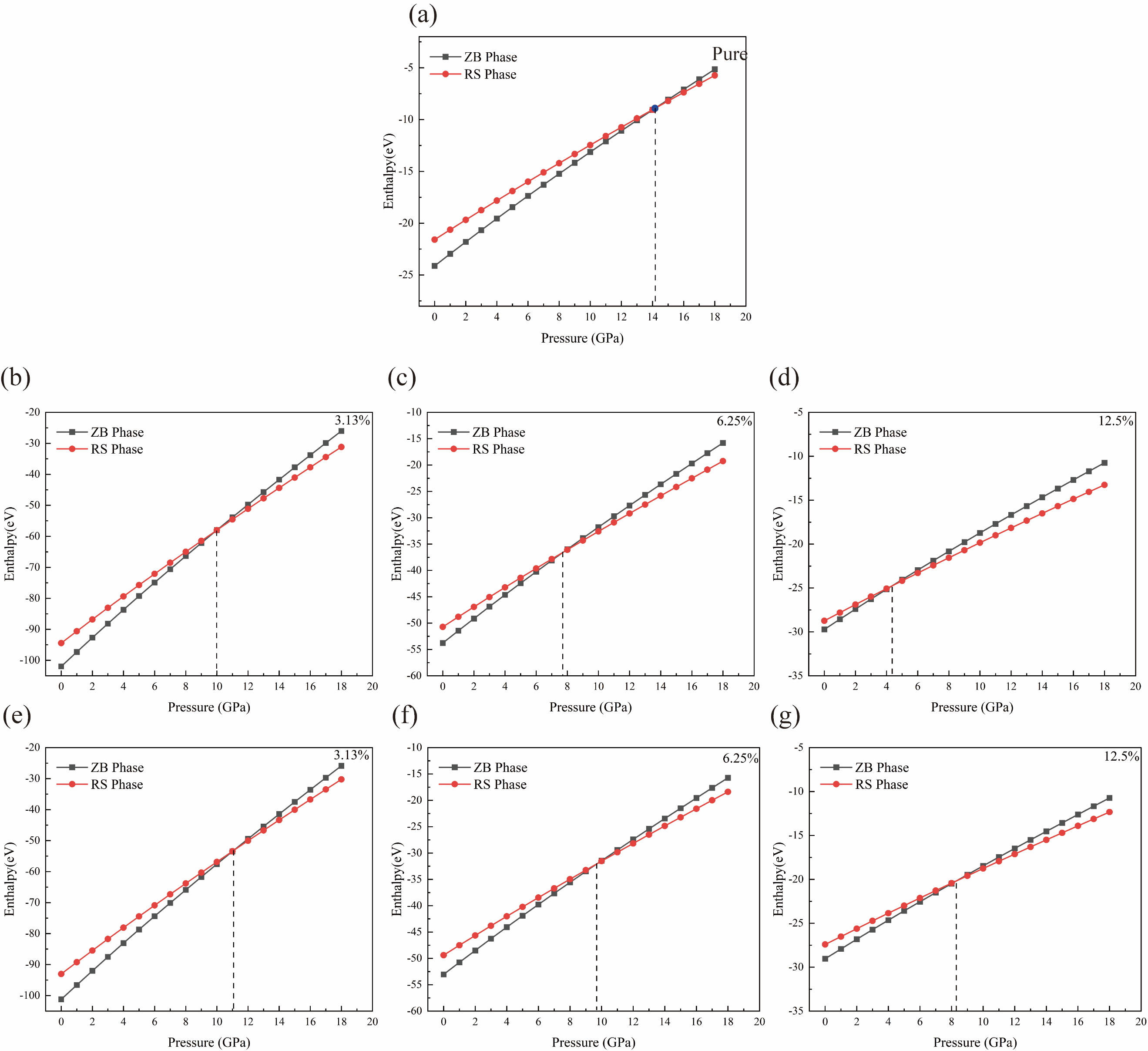


**Supplementary Figure 1.** Pure ZnSe and V/Mn:ZnSe enthalpy and pressure curve(a)Pure ZnSe; (b)-(d)V:ZnSe;(e)-(g) Mn:ZnSe.

## Magnetic analysis

When the transition metals V and Mn are doped with II-VI semiconductors, V^2+^ and Mn^2+^ have 3 and 5 unpaired d electrons, respectively. Therefore, the theoretical transition metal ions will have a magnetic moment of 3µ_B_ and 5µ_B_, respectively. Supplementary Table 1 provides the total magnetic moment M_tot_ of the system doped with ZnSe by V and Mn and the magnetic moment of the doped atoms M_doped_. As shown in Supplementary Table 1, pure ZnSe is nonmagnetic at both atmospheric and high pressures. The ionic magnetic moments embodied in each doped system are smaller than the theoretical values, which, according to the previous experience^1^, is due to the full filling of the spin-up t_2g_ orbitals of the doped transition metals. Supplementary Fig. 2. shows the spin charge density plots of the doped systems. It can be seen that the magnetic moments in both systems are mainly localized on the dopant atoms, and the spin densities on the Zn and Se atoms are almost zero, which agrees with the calculations in Supplementary Table 1. Due to the formation of a more stable, fully filled shell configuration, Se makes a very small contribution to the magnetic moment. It can be seen from the total magnetic moment of the doping system that when the atomic number increases, the corresponding total magnetic moment also increases. This is mainly because the atomic radius of the transition metals V and Mn gradually decreases, so that the force of the nucleus on the outer shell electrons gradually increases, resulting in the weakening of the activity of the electrons in the d layer and the increasing number of electrons in the d layer. The magnetic strength of the doped system is mainly determined by the 3d electronic states of the transition metals V and Mn, so the total magnetic moment of the doped system gradually increases with the increase of the number of electrons in the d layer.

Charge transfer theory^2^ can also explain the source of magnetic properties of the system. In order to obtain a stable Se^2-^ state, each Se atom has to get two electrons from the nearest Zn atom or doped transition metal (V/Mn). In pure ZnSe, Zn atom loses two valence electrons to neighboring Se atom, so Zn ion shows +2 valence state and corresponding Se ion shows -2 valence state, Zn^2+^ ion has 3d^8^ valence electrons, and after orbital hybridization electron rearrangement, all d electrons are paired. According to the Pauli exclusion principle pairs of electrons in the same orbital have opposite spin directions, so no local magnetic moments can be generated in pure ZnSe, so it is not magnetic. When a V or Mn atom replaces a Zn atom, it also transfers two valence electrons to the Se atom, leaving three and five 3d electrons, respectively. The remaining 3d electrons on the transition metal atoms split further, but are not necessarily fully polarized due to non-integer magnetic moments^3^. The results of Bader charge analysis show that the net Bader charges on the doped atoms are 11.80e, 11.74e and 11.73e (12.21e, 12.14e and 12.22e) when the doping amount of V (Mn) is 3.13%, 6.25% and 12.5%, respectively, and the Bader charges of Se atom are 7.0e, 6.97e and 6.99e (6.56e, 6.73e and 6.68e). It indicates that there is a strong charge transfer between the doped atom and Se atom, and electrons are transferred from the doped atom to the Se atom, which is different from that of pure ZnSe. Therefore, the V or Mn doped ZnSe system is magnetic. In addition, it can be seen from Supplementary Table 1 that the overall total magnetic moment of the V: ZnSe system at high pressure is slightly larger than that at atmospheric pressure, while the local magnetic moment of the V atoms is slightly smaller than that at atmospheric pressure. The total magnetic moment of Mn:ZnSe at high pressure and the local magnetic moment of doped Mn atoms are both slightly smaller than that at atmospheric pressure, and the overall change of the total and local magnetic moments by pressure is not obvious. This implies that pressure does not have a significant effect on the magnetic properties of the V/Mn:ZnSe system, and this discovery could broaden its application as a stabilizing magnetic device in high-pressure environments.


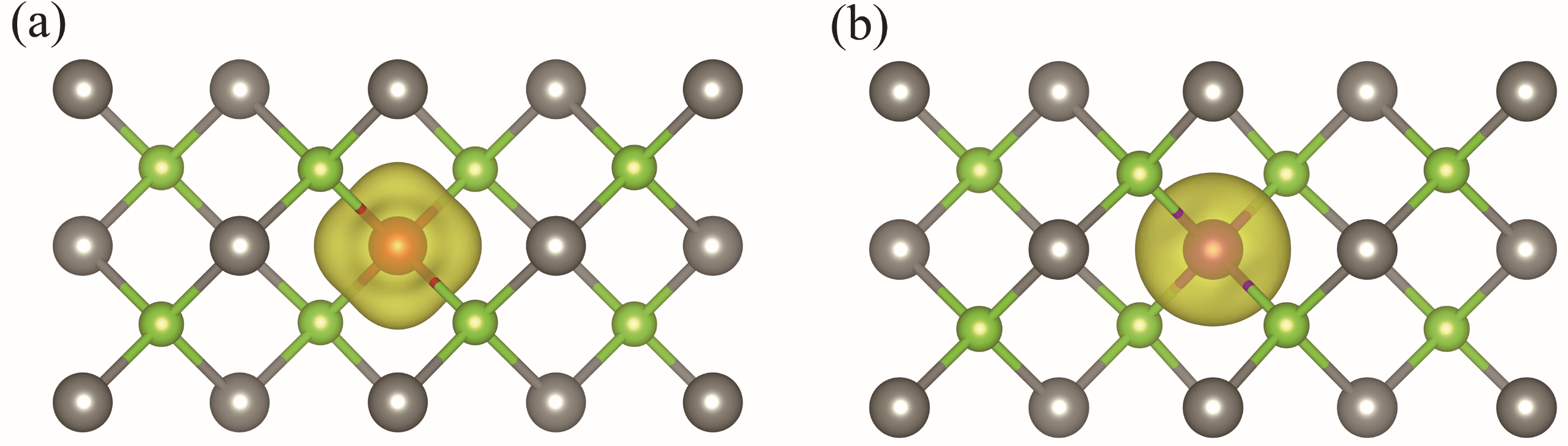


**Supplementary Figure 2. Spin charge density (a) V:ZnSe; (b) Mn:ZnSe.**

**Supplementary Table 1**. Magnetic moments of the system after V or Mn doping of ZnSe.

|  | M_tot_ (µ_B_) | | M_doped_ (µ_B_) | | M_Se_ (µ_B_) | |
| --- | --- | --- | --- | --- | --- | --- |
|  | 0GPa | High pressure | 0GPa | High pressure | 0GPa | High pressure |
| Pure ZnSe | 0 | 0 | / | / | / | / |
| V-doped |  |  |  |  |  |  |
| 3.13% | 2.394 | 2.415 | 2.334 | 2.304 | -0.026 | -0.017 |
| 6.25% | 2.395 | 2.416 | 2.351 | 2.330 | -0.034 | -0.026 |
| 12.5% | 2.431 | 2.421 | 2.392 | 2.369 | -0.008 | -0.006 |
| Mn-doped |  |  |  |  |  |  |
| 3.13% | 4.350 | 4.323 | 3.999 | 3.848 | 0.027 | 0.043 |
| 6.25% | 4.351 | 4.327 | 4.001 | 3.872 | 0.038 | 0.052 |
| 12.5% | 4.355 | 4.336 | 4.013 | 3.910 | 0.044 | 0.056 |

1 Kacman, P. Spin interactions in diluted magnetic semiconductors and magnetic semiconductor structures. *Semiconductor Science and Technology* **16**, R25, doi:10.1088/0268-1242/16/4/201 (2001).

2 Chen, Q. & Wang, J. Structural, electronic, and magnetic properties of TMZn_11_O_12_ and TM_2_Zn_10_O_12_ clusters (TM=Sc, Ti, V, Cr, Mn, Fe, Co, Ni, and Cu). *Chemical Physics Letters* **474**, 336-341, doi:<https://doi.org/10.1016/j.cplett.2009.05.006> (2009).

3 Cao, J., Cui, L. & Pan, J. Magnetism of V, Cr and Mn doped MoS_2_ by first-principal study. *Acta Physica Sinica* **62**, 187102-187102, doi:10.7498/aps.62.187102 (2013).
